# Mapping the Missing: Assessing Amphibian Sampling Completeness and Overlap With Global Protected Areas

**DOI:** 10.1002/ece3.71137

**Published:** 2025-05-05

**Authors:** Jorge Mario Herrera‐Lopera, Mirco Solé, Carlos A. Cultid‐Medina

**Affiliations:** ^1^ Programa de Pós‐Graduação Em Ecologia e Conservação da Biodiversidade Universidade Estadual de Santa Cruz (UESC) Ilhéus Brazil; ^2^ Tropical Herpetology Lab, Departamento de Ciências Biológicas Universidade Estadual de Santa Cruz (UESC) Ilhéus Brazil; ^3^ Grupo de Investigación en Biodiversidad y Recursos Naturales (BIONAT), Semillero de Investigación en Biodiversidad y Conservación de Paisajes Urbanos (OIKOS) Universidad de Caldas Manizales Colombia; ^4^ Departamento de Ciências Biológicas Universidade Estadual de Santa Cruz (UESC) Ilhéus Brazil; ^5^ Museum Koenig Bonn (ZFMK) Leibniz Institute for the Analysis of Biodiversity Change Bonn Germany; ^6^ Red de Diversidad Biológica del Occidente Mexicano Instituto de Ecología, A. C., Centro Regional del Bajío Mexico; ^7^ SECIHTI Ciudad de México Mexico

**Keywords:** amphibian conservation, distribution data gaps, key biodiversity areas, natural protected areas, research prioritization, sampling completeness

## Abstract

The aim of the study was to assess amphibian sampling completeness and the overlap of sampling completeness categories with natural protected areas (NPAs) and key biodiversity areas (KBAs) at global scale. We evaluated amphibian sampling completeness across six of the earth's eight biogeographic realms to identify *well‐sampled*, *under‐sampled*, and *data‐gap* areas in the context of global amphibian distribution. Additionally, we examined the spatial overlap of each sampling category with NPAs and KBAs. The Nearctic and Australasian realms had the highest number of records and well‐sampled areas. Significant data gaps were identified, particularly in the Afrotropical, Indo‐Malayan, Neotropical, and Palearctic realms. We found low levels of spatial match (< 35%) between classified areas and NPAs/KBAs. Amphibian distribution data are largely incomplete, with the most extensive gaps in the most species‐rich realms: Neotropic, Indo‐Malayan, and Afrotropical. The low overlap between under‐sampled and data‐gap areas with NPAs and KBAs suggests that these regions, critical for amphibian diversity, are insufficiently represented within established conservation priorities. Given the urgent threats to biodiversity from global change, rapid responses are essential to enhance our understanding of species distributions and community structures in amphibians. This study provides spatial insights to help identify key data‐gap areas for amphibian research and conservation prioritization.

## Graphical abstract

This study evaluates global sampling completeness of amphibians and explores the overlap between sampling categories and conservation areas, focusing on natural protected areas (NPAs) and key biodiversity areas (KBAs). Results reveal significant data gaps, particularly in the most species‐rich realms, and a low spatial match with NPAs/KBAs. These findings underscore the need for targeted data collection to improve amphibian knowledge and lead conservation efforts.

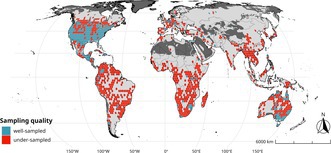

## 1 Introduction

Amphibians are the most threatened vertebrate group, with more than 40% of their species under some threat category (IUCN 2024a; Luedtke et al. 2023; da Silva et al. 2020). Habitat loss, emerging diseases, climate change, and interactions among these factors are the main causes of the global decline in amphibian populations (Wake 1991; Wake and Vredenburg 2008). In addition, although amphibians are one of the groups for which most human and economic capital is allocated (Cardoso et al. 2011; however, see IUCN SSC 2022), knowledge about many basic aspects of amphibian biology is incipient. For example, between 2010 and 2020, about 20% of the currently recognized species were described, and the rate of description continues to increase (Frost 2024—Comparing amphibian richness of 5.3 and 6.2 versions). There is also a taxonomic bias in the ecological knowledge of amphibians (da Silva et al. 2020): for most species, aspects such as responses to environmental pressures, natural history, and distribution are still unknown (Nori et al. 2023).

Understanding how and where species are distributed is fundamental in order to formulate efficient strategies for their conservation at a global scale (Mi et al. 2023; Nori et al. 2023; Serrano et al. 2023). However, the knowledge available on amphibian distribution is taxonomically and geographically biased, under‐representing species with cryptic habits (e.g., fossorial or canopy‐dwelling species) while reporting a higher number of records for more conspicuous species (Hughes et al. 2021; Martin et al. 2012; da Silva et al. 2020). Additionally, most records across biological groups are concentrated mainly in North America and Europe, despite the highest species richness being found in tropical regions (Girardello et al. 2019; Hughes et al. 2021; Martin et al. 2012; Pelayo‐Villamil et al. 2018; Vitt and Caldwell 2014). The recognition of this geographic bias allows the identification of priority areas for directing research resources. Campbell Grant et al. (2023) emphasized an essential aspect of amphibian conservation: the urgent need to determine both the number and identity of species protected within natural protected areas (NPAs). With human impacts increasingly altering natural systems, NPAs serve as the primary approach for in situ biodiversity conservation. Furthermore, in the face of global change, NPAs are expected to act as “*stepping stones*” supporting species as they navigate future environmental challenges (Thomas and Gillingham 2015; Watson et al. 2016, 2014). Mi et al. (2023) found that most amphibian species were recorded in at least one NPA. However, having a record within an NPA does not guarantee effective conservation for a species, since large portions of their distribution range remain unprotected (Mi et al. 2023) or unknown, and it is uncertain whether the populations within NPAs are viable for long‐term survival. Therefore, it is essential to understand the completeness of our current knowledge to accurately assess the “actual distribution” of amphibian species (i.e., distribution based on specimen records rather than inferred from the area of occurrence or areas of occupancy) and their presence within protected areas. This clarity will also help prioritize efforts to fill information gaps, refining our ability to make informed conservation decisions.

Sampling completeness is understood as the ratio between observed species and expected species for a region and is a useful tool to determine areas where it is necessary to increase sampling effort (Chao et al. 2020; Lobo et al. 2018), in this case, to improve efforts to collect data related to amphibian distribution. Assessing and mapping areas with low data completeness and significant information gaps on a global scale aligns with the goals of the Convention on Biological Diversity (Convention on Biological Diversity 2010) and its “2030 Targets” under the Kunming‐Montreal Global Biodiversity Framework (KMGBF), to which over 200 countries are committed (Kunming‐Montreal Global Biodiversity Framework 2022; Plumptre et al. 2024). One key target of this framework aims to “conserve 30% of land, waters, and seas” by 2030, serving as an update to the Aichi Targets (Butchart et al. 2015; Convention on Biological Diversity 2010; Plumptre et al. 2024; Santini et al. 2016). Providing spatially explicit data on areas lacking amphibian distribution information (i.e., regions with low data completeness or no data at all) can greatly support decision‐makers in directing resources to priority research areas. This targeted research will help determine whether these areas are suitable as potential expansion and connectivity zones for existing NPAs worldwide. Additionally, key biodiversity areas (KBAs) complement NPAs by identifying critical ecosystems that support vulnerable populations and should be preserved due to their ecological significance and integrity (IUCN 2016). Although KBAs are not officially classified as NPAs, they play a crucial role in site‐based conservation efforts led by governments and the private sector (IUCN 2016; Plumptre et al. 2024). Identifying gaps in amphibian distribution data can help refine the designation of KBAs and therefore strengthen site‐based conservation initiatives.

The Global Biodiversity Information Facility (GBIF) is the most comprehensive open database of biological records on a global scale (Girardello et al. 2019), besides the IUCN spatial data. The difference between GBIF and IUCN spatial data is that the former consists mainly of records in georeferenced points format, while the IUCN provides distribution polygons for species, which are often interpretations of GBIF data interpreted by experts (Boitani et al. 2011). Therefore, GBIF data are likely to have fewer co‐omission errors than IUCN data, and IUCN data may underestimate areas with gaps in distribution information by assuming species distributions in areas where no data are available—due to the use of polygons (IUCN 2024b). The GBIF brings records from different sources, such as articles, museum collections, and citizen records, among others. Some authors have proposed that records in the GBIF (especially those coming from citizens' contributions) might exhibit misidentification issues and bias: areas of higher human population density correlating with the number of species records (Girardello et al. 2019; Hughes et al. 2021; Shirey et al. 2021). The use of GBIF databases for ecological research has also been criticized because they combine information from different sources with a sampling effort that is not comparable with each other (see Lobo 2008). However, the approaches of Lobo (2008) and Lobo et al. (2018) allow the use of this heterogeneous source resource as raw material for estimating completeness and identifying areas with information gaps and areas without information in simple and comparable units.

This study aimed to assess and pinpoint areas worldwide characterized by information gaps in the distribution of amphibians, highlighting the situation for each of the biogeographical realms. Our questions were: (i) where are the well‐sampled, under‐sampled, and data‐gap areas for amphibian distribution globally? and (ii) what level of spatial coincidence do well‐sampled, under‐sampled, and data‐gap areas have with currently designated NPAs and KBAs on a global scale? We aim to provide valuable, clear, and spatially explicit information that identifies areas with data gaps in the global understanding of amphibian distribution.

## 2 Methods

### 2.1 Data Regionalization

We follow Olson et al. (2001) biogeographic proposal modified by Dinerstein et al. (2017), which divides the globe into 8 biogeographic realms and 14 biomes. These realms are: Afrotropic, Antarctic, Australasian, Indo‐Malayan, Nearctic, Neotropic, Oceania, and Palearctic. To select a biogeographic framework, we compared this approach with that of Holt et al. (2013) and found that both frameworks are equally suitable for dividing amphibian distributions at a global scale (see Supporting Information S1). However, we chose Olson et al. (2001) and Dinerstein et al. (2017) because they allow for the analysis of information gaps at the biome level. Although a biome‐level analysis is not one of our primary objectives, we included it as an additional analysis in Supporting Information S2. It is important to note that the Antarctic and Oceania realms were excluded from the study, as amphibians are absent in the Antarctic and most oceanic islands lack amphibian species (Vitt and Caldwell 2014) and that just a small portion of the records deposited in GBIF, after data curation, were from the Oceania realm (0.03%, ~75 records).

We excluded areas without amphibian distribution data according to the IUCN spatial data (IUCN 2024c) to avoid overestimating information gap areas. While some GBIF records fall within these excluded regions, aligning our analysis with the expert‐informed IUCN distribution polygons provides a standardized approach. Although this method may introduce expert bias, potentially underestimating information gaps by inferring species presence in areas lacking explicit records, it offers a uniform basis for delineating species ranges and identifying unrepresented regions.

### 2.2 Amphibian Global Occurrence Records

We utilized amphibian records from the GBIF and the *rgbif* package in R to extract records associated with museum vouchers, thereby minimizing the potential for misidentification (Chamberlain et al. 2023; Daru and Rodriguez 2023; GBIF.Org 2024; R Core Team 2023). The records were curated using functions of the *scrubr* and *CoordinateCleaner* R packages (Chamberlain 2022; R Core Team 2023; Zizka et al. 2019). Given the heterogeneity of GBIF data sources and the lack of assurance that they originate from surveys with comparable sampling effort, duplicate records—defined as entries for the same species at identical latitude–longitude coordinates—were removed unless they represented different collection years, since we used collection years as surrogates in the estimation of sampling completeness (Lobo 2008; Lobo et al. 2018). Taxonomy of records was updated and corrected using the functions and databases stored in the *AmphiNom* R package (Liedtke 2019; R Core Team 2023), which is grounded in the “Amphibian Species of the World 6.2” database from the American Museum of Natural History (Frost 2024). Taxonomic information is updated to April 2024. Given the large number of records used in this study, and because species distribution polygons do not necessarily align with the available GBIF records, we aimed to minimize misidentified points by performing the data curation steps described above.

### 2.3 Natural Protected Areas and Key Biodiversity Areas

The polygons for NPAs were obtained from The World Database on Protected Areas (WDPA—August 2023 version), which comprises the largest global database of terrestrial and marine NPAs. The database includes national and international conservation areas defined by governments as well as other effective area‐based conservation measures (OECMs). Thus, National Parks, Habitat Reserves, Indigenous Reserves, and Ramsar Areas, among many others, are also considered. The polygons can be freely downloaded from protectedplanet.net (see UNEP‐WCMC, IUCN 2023).

The KBAs were obtained upon request from www.keybiodiversityareas.org (BirdLife International 2024); we used the March 2024 version.

### 2.4 Data Analysis

To evaluate the amphibian sampling completeness at the global level, we used the *KnowBR* package of the R language (Lobo et al. 2018; R Core Team 2023). The package has the advantage of allowing the use of records collected with different methodological approaches for the completeness estimation, as is the case with GBIF data, which comes from different types of studies (Lobo 2008; Lobo et al. 2018; Pelayo‐Villamil et al. 2018). The package requires “event – independent” records of the species as “surrogates” for the calculation of completeness (see Lobo 2008). Therefore, we considered records with different collection years (column “year” in the GBIF data) as event‐independent data.

To assess completeness levels within each realm, we divided each realm into hexagonal polygons with a width of 2° (~222.64 km at equator) and calculated completeness for each polygon. Although we considered other resolutions, our analysis revealed a low effect size of hexagon size on completeness (*ω* ^2^ = 0.007; see Supporting Information S3). Consequently, we opted to use the 2° resolution, as it maximizes species richness while minimizing species co‐omission.

For the calculation of completeness through the *KnowBR* package, we used the following parameters: (i) rational curve because we had no basis for assuming an established model of density distribution or accumulation curve for amphibians, (ii) exact Ugland et al. (2003) estimator, since we were interested in a precise estimate rather than the variation intervals in the estimate, and (iii) a minimum ratio of 1 (“*cutoff*” in the function) to ensure that there were more records than species within each of the hexagons (Lobo et al. 2018; Pelayo‐Villamil et al. 2018).

To understand the variation in sampling completeness among realms, we compared the confidence intervals of completeness for each realm (Cumming et al. 2007).

Lobo et al. (2018) and Pelayo‐Villamil et al. (2018) recommended classifying the sampling completeness values into three categories to get the survey quality: (i) *poor*, when sampling completeness is < 50%, the ratio between species and records is < 3 and the slope value in the estimated accumulation curve is > 0.3, (ii) *good*, when sampling completeness is > 90%, the ratio between species and records is > 15 and the slope value in the estimated accumulation curve is < 0.02, and (iii) *fair*, for values in the middle. Since these values are defined by default and in the majority of studies where these criteria have been applied are at administrative division scale, where criteria such as the ratio between records and species can be more easily achieved than in cells of smaller size, we decided to modify the criteria. We adjusted them according to the proposals of Yang et al. (2013) and Girardello et al. (2018). They justify their classification criteria on the slope value of the estimated accumulation curve, since the slope is a reflection of the saturation of the curve and can be interpreted as the “probability of discovering new species when sampling continues”; therefore, the slope should be a more accurate classification criterion than the sampling completeness value itself. They consider a slope value ≤ 0.05 as “well sampled” and slopes > 0.05 as “under‐sampled”. Thus, we decided to define three categories: (i) “*well‐sampled*”, with slope ≤ 0.05, (ii) “*under‐sampled*”, with slope > 0.05, and (iii) “*data‐gap*”, for areas where species records should exist according to the IUCN (2024b) spatial data, but where sampling completeness could not be calculated due to insufficient records, (i.e., record‐to‐species ratio of ≤ 1).

To assess the percentage of spatial congruence between the classified zones and the NPAs and KBAs for each realm, we calculated the percentage of spatial match (Nori et al. 2020). This was done by overlaying the vector layers, calculating the overlapping area, and then dividing this by the total area for each classified zone. The analysis was carried out using *terra* and *tyditerra* R packages (Hernangómez 2023; Hijmans 2024; R Core Team 2023). All calculations were performed using a global equal‐area projection (Eckert IV, ESRI:54012) to preserve the true size of spatial features (i.e., hexagons, NPAs, and KBAs) worldwide.

## 3 Results

### 3.1 Records

**FIGURE 1:**
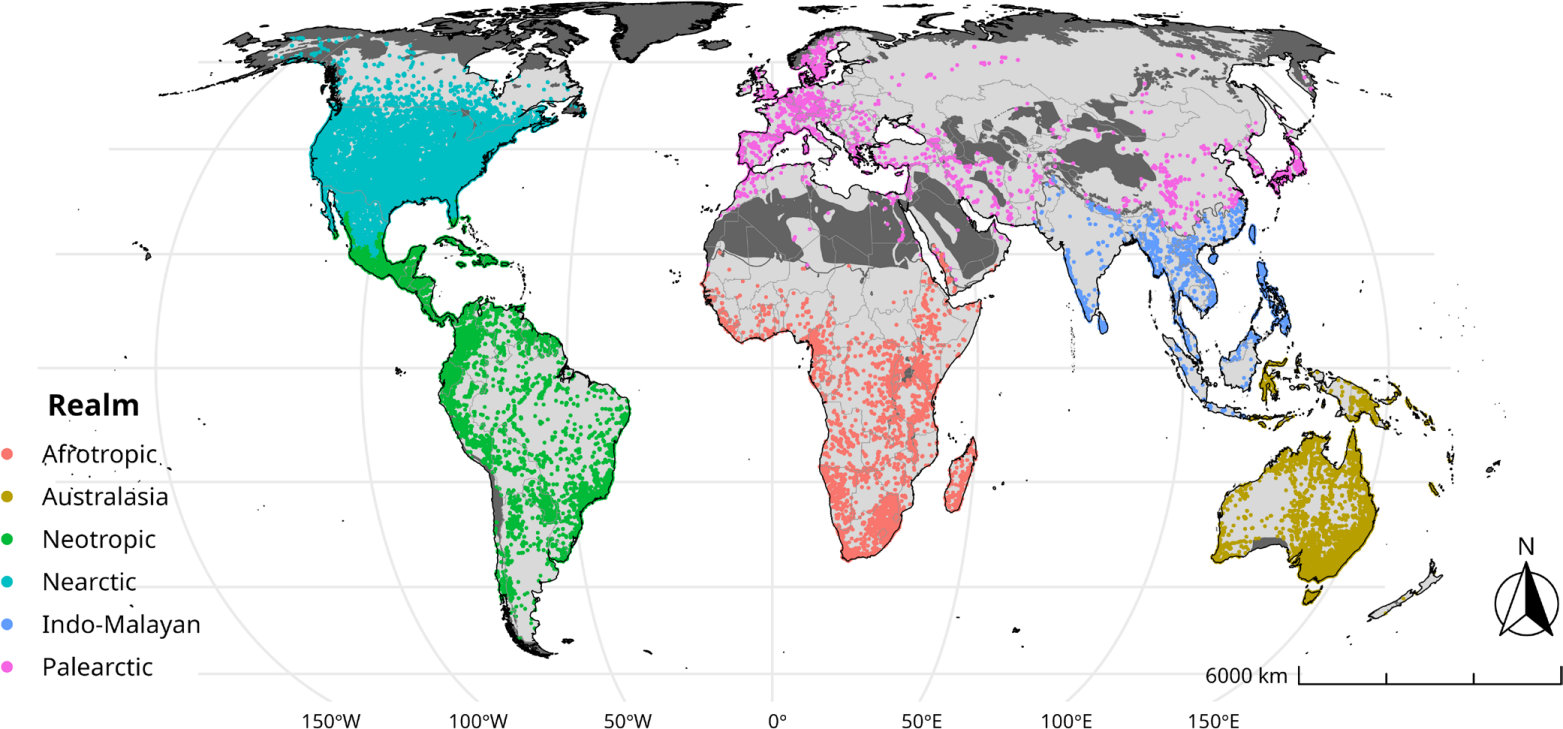
Curated GBIF amphibian records. Dark gray shows non‐included areas due to the absence of amphibian species distribution, according to the IUCN (2024b) spatial data. Light gray represents the geographic extent of this work lacking records. Datum: Eckert IV projection, EPSG:54012.

A total of 2,239,932 occurrence records were downloaded and curated from the GBIF. After curating the data, we retained 246,384 unique locality‐year records across 73 families, 509 genera, and 4891 species. The Nearctic realm contained most of the records (52.8%), followed by the Neotropical (23.9%) and Australasian realms (10.1%), while each of the remaining realms accounted for less than 10% (Figure 1, and see precise numbers in Table 1).

**TABLE 1:** Records, species, genera, and families of amphibians included in this study by realm.

Realm	Records number (%)	Species number (%)	Genera number (%)	Families number (%)
Afrotropic	17,558 (7.13)	819 (16.75)	104 (20.43)	22 (30.14)
Australasia	24,841 (10.08)	384 (7.85)	53 (10.41)	10 (13.7)
Indo‐Malayan	8247 (3.35)	662 (13.54)	104 (20.43)	17 (23.29)
Nearctic	130,204 (52.85)	419 (8.57)	74 (14.54)	26 (35.62)
Neotropic	58,871 (23.89)	2594 (53.04)	228 (44.79)	40 (54.79)
Palearctic	6663 (2.7)	275 (5.62)	80 (15.72)	19 (26.03)
Total	246,384 (100)	4891 (100)	509 (100)	73 (100)

### 3.2 Sampling Completeness

The proportion of the area for which sampling completeness could be estimated (i.e., under‐sampled and well‐sampled areas) varied across realms. In the Nearctic realm, sampling completeness could be calculated for 65% of the area, followed by the Australasian realm (63%) and the Neotropical realm (55%). The Palearctic realm had the lowest proportion of area with estimated completeness, at only 12%. Consequently, the Palearctic was the realm with the largest amount of data gaps, with 88% of its area within this category (Table 2).

**TABLE 2:** Proportion of areas in each sampling quality category for amphibian distributions across realms.

Realm	Total area	Ws area (%)	Us area (%)	Dg area (%)
Afrotropic	2164.56	53.92 (2.49)	909.59 (42.02)	1201.05 (55.49)
Australasia	904.95	188.27 (20.8)	381.87 (42.2)	334.81 (37)
Indo‐Malayan	848.39	12.36 (1.46)	325.04 (38.31)	510.99 (60.23)
Nearctic	1576.08	627.5 (39.81)	402.69 (25.55)	545.89 (34.64)
Neotropic	1887.44	128.72 (6.82)	909.03 (48.16)	849.69 (45.02)
Palearctic	3809.94	81.07 (2.13)	379.7 (9.97)	3349.17 (87.91)

*Note:* Areas are presented in million hectares (Mha). Percentages were calculated as the area of each category divided by the total area of each realm, multiplied by 100.

Abbreviations: Dg, data‐gap; Us, under‐sampled; Ws, well‐sampled.

**FIGURE 2:**
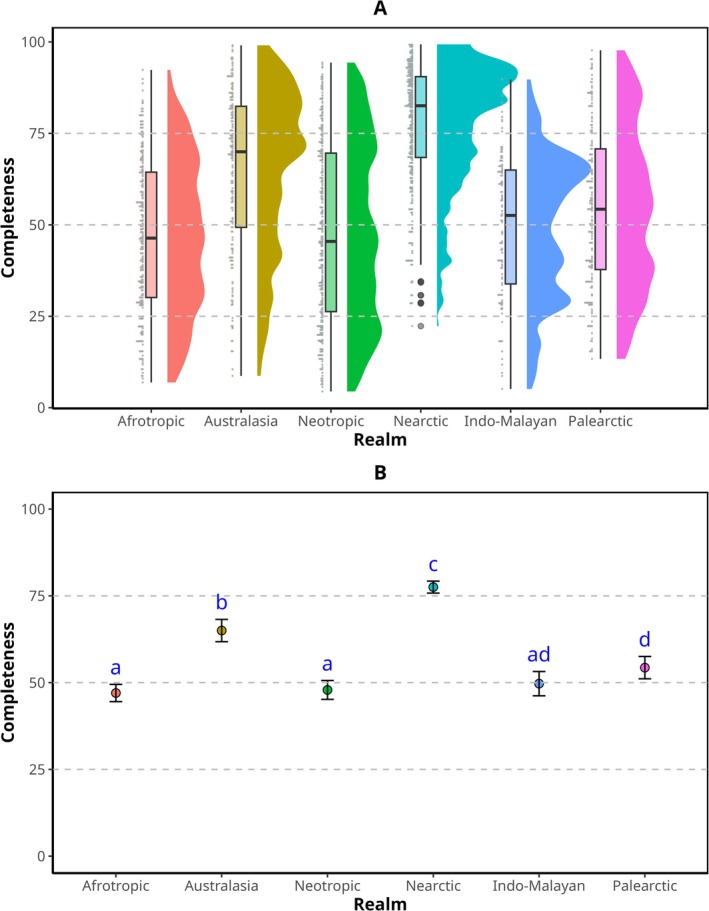
Estimated sampling completeness for amphibian distribution between realms. (A) Density distribution of completeness values. (B) Mean and 95% confidence intervals of completeness between realms; different letters represent a non‐overlap of the confidence intervals.

The average completeness for hexagons differed between realms. The Nearctic realm (mean: 77.5%, 95% CI: 75.8%–79.2%) and Australasia (mean: 65%, 95% CI: 61.8%–68.2%) exhibited the highest levels of average sampling completeness. In contrast, the realms with the lowest average completeness were Afrotropical (mean: 47%, 95% CI: 44.5%–49.5%), Indo‐Malayan (mean: 49.7%, 95% CI: 46.2%–53.2%), and Neotropical (mean: 47.9%, 95% CI: 45.2%–50.6%). The Palearctic realm (mean: 54.3%, 95% CI: 51.1%–57.5%) showed significantly lower average completeness than the Nearctic and Australasia but higher than the Afrotropical and Neotropical, and not significantly different from the Indo‐Malayan realm (Figure 2 and Supporting Information S4).

**FIGURE 3:**
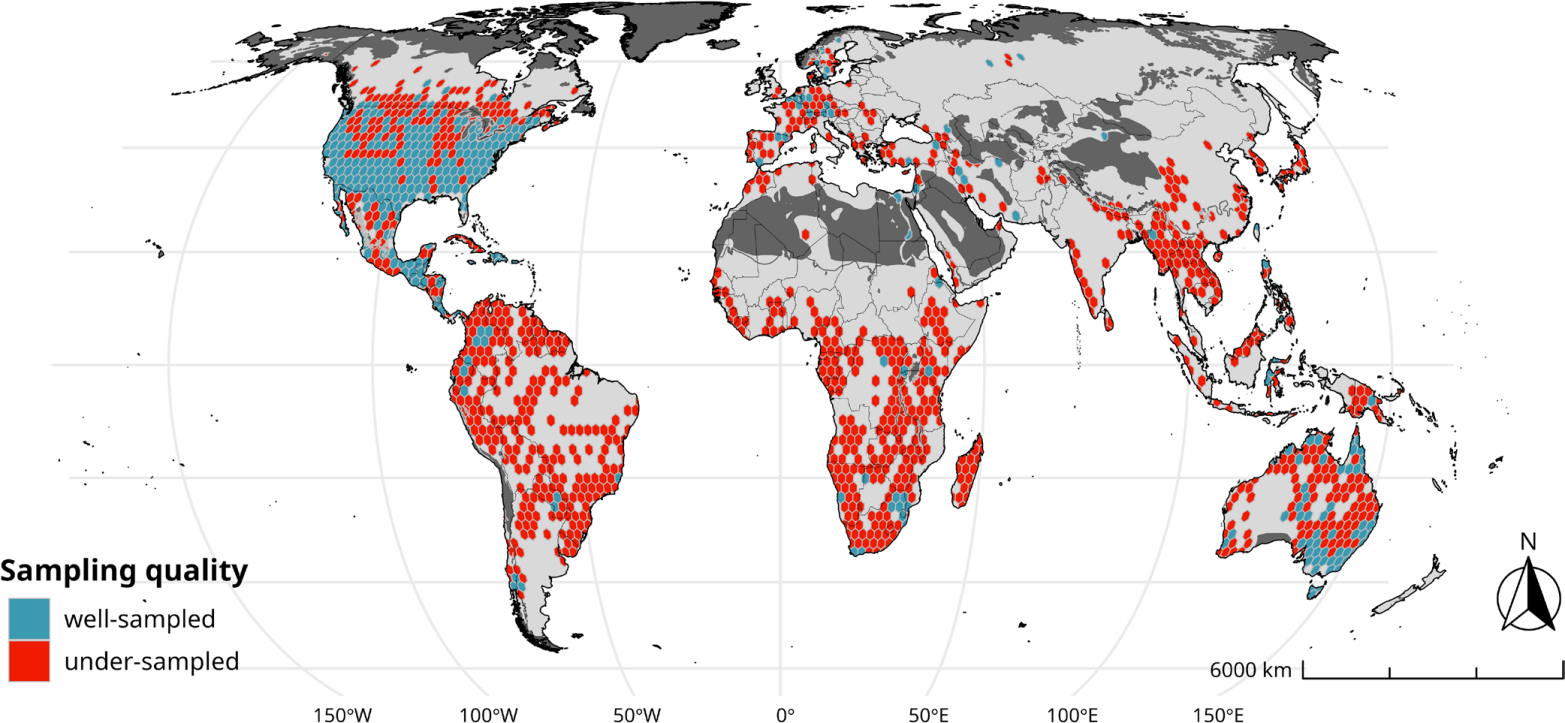
Sampling quality of amphibians at a global scale. Dark gray areas indicate regions excluded because no amphibian species distributions are expected there according to the (IUCN 2024c) spatial data. Light gray represents the geographic extent of this work where completeness could not be estimated and are thus classified as data‐gap. Datum: Eckert IV projection, EPSG:54012.

The Nearctic realm had the highest proportion of well‐sampled areas (ws: 0.4, us: 0.26, dg: 0.35), followed by Australasia (ws: 0.21, us: 0.42, dg: 0.37) and Neotropic (ws: 6.82, us: 48.16, dg: 45.02). In these three realms, the combined proportion of well‐sampled and under‐sampled areas exceeded that of data‐gap areas. For all other realms, the proportion of data‐gap areas was greater, indicating that most of the realms lacked sufficient information to make reliable calculations of sampling completeness (Table 2, Figure 3, and Supporting Information S4).

### 3.3 Overlap With NPAs

The overlap of well‐sampled with NPAs ranged from 11% to 32%, with the highest percentages observed in the Neotropic (32.47%), Afrotropic (28.38%), and Palearctic (25.47%) realms. For under‐sampled areas, the overlap ranged from 10% to 29%, with the highest percentages in the Neotropic (29.41%), Afrotropic (25.24%), and Palearctic (20.47%) realms. For data‐gap areas, the overlap ranged from 6% to 33%, with the highest percentages in the Neotropic (33.28%), Australasia (27.19%), and Afrotropic (18.06%) realms (Table 3).

**TABLE 3:** Overlap between well‐sampled, under‐sampled, and data‐gap areas for amphibian distribution across realms and the natural protected areas.

Realm	Total area	NPAs area (%)	Ws area	Us area	Dg area	Overlap with natural protected areas (NPAs)		
						Ws area (%)	Us area (%)	Dg area (%)
Afrotropic	2164.56	461.82 (21.34)	53.92	909.59	1201.05	15.3 (28.38)	229.58 (25.24)	216.94 (18.06)
Australasia	904.95	196.24 (21.69)	188.27	381.87	334.81	44.86 (23.83)	60.35 (15.8)	91.03 (27.19)
Indo‐Malayan	848.39	72.81 (8.58)	12.36	325.04	510.99	1.63 (13.19)	39.44 (12.13)	31.74 (6.18)
Nearctic	1576.08	203.24 (12.9)	627.50	402.69	545.89	67.44 (10.75)	40.12 (9.96)	95.68 (17.52)
Neotropic	1887.44	591.83 (31.36)	128.72	909.03	849.69	41.79 (32.47)	267.33 (29.41)	282.71 (33.28)
Palearctic	3809.94	495.8 (13.01)	81.07	379.70	3349.17	20.65 (25.47)	77.74 (20.47)	397.41 (11.87)

*Note:* Areas expressed in million hectares (Mha). NPA percentages represent the proportion of NPA area within each realm relative to the realm's total area, multiplied by 100. Category percentages reflect the overlap of each sampling category with NPAs, calculated by dividing the overlap area by the total area of that category within each realm and multiplying by 100.

Abbreviations: Dg, data‐gap; Us, under‐sampled; Ws, well‐sampled.

### 3.4 Overlap With KBAs

The Neotropical, Afrotropical, and Indo‐Malayan realms showed the highest overlap across all categories, with well‐sampled areas ranging from 4% to 27% (highest in Neotropical, 26.93%), under‐sampled areas from 4% to 16% (highest in Neotropical, 15.58%), and data‐gap areas from 3% to 13% (highest in Neotropical, 12.68%). The Palearctic realm also ranked high in the under‐sampled category (15.07%) (Table 4).

**TABLE 4:** Overlap between well‐sampled, under‐sampled, and data‐gap areas for amphibian distribution across realms and the key biodiversity areas.

Realm	Total area	KBAs area (%)	Ws area	Us area	Dg area	Overlap with key biodiversity areas (KBAs)		
						Ws area (%)	Us area (%)	Dg area (%)
Afrotropic	2164.56	250.48 (11.57)	53.92	909.59	1201.05	12.67 (23.5)	121.34 (13.34)	116.47 (9.7)
Australasia	904.95	65.72 (7.26)	188.27	381.87	334.81	24.78 (13.16)	23.22 (6.08)	17.72 (5.29)
Indo‐Malayan	848.39	90.31 (10.64)	12.36	325.04	510.99	2.4 (19.42)	43.28 (13.32)	44.63 (8.73)
Nearctic	1576.08	55.8 (3.54)	627.50	402.69	545.89	23.46 (3.74)	16.23 (4.03)	16.11 (2.95)
Neotropic	1887.44	284.01 (15.05)	128.72	909.03	849.69	34.67 (26.93)	141.62 (15.58)	107.72 (12.68)
Palearctic	3809.94	335.57 (8.81)	81.07	379.70	3349.17	11.4 (14.06)	57.22 (15.07)	266.95 (7.97)

*Note:* Areas are expressed in million hectares (Mha). KBA percentages represent the proportion of KBA area within each realm relative to the realm's total area, multiplied by 100. Category percentages reflect the overlap of each sampling category with KBAs, calculated by dividing the overlap area by the total area of that category within each realm and multiplying by 100.

Abbreviations: Dg, data‐gap; Us, under‐sampled; Ws, well‐sampled.

## 4 Discussion

Our results indicate that amphibian sampling quality at the global level is geographically biased, and that greater efforts are needed to inventory amphibians in regions with the highest reported species richness. The Nearctic and Australasia realms had the highest proportion of well‐sampled areas, while the Neotropical, Indo‐Malayan, and Afrotropical realms had the highest proportion of under‐sampled areas. Data‐gap areas were larger in the Palearctic, Indo‐Malayan, and Afrotropical realms (Question i). Overall, the spatial congruence of all areas (i.e., well‐sampled, under‐sampled, and data‐gap) with the NPAs was low, not exceeding 35% in any realm (Question ii). Similarly, overlap with the KBAs was generally low, with well‐sampled areas showing the highest overlap values, and only the Neotropic realm slightly exceeding 25% (Question 2).

Our results are based on the distribution data for 93.6% of families (73 out of 78), 89.5% of genera (509 out of 569), and 56.2% of species (4891 out of 8708) of amphibians worldwide (Frost 2024; Liedtke 2019). Although our representativeness is relatively low compared to other ecological studies with amphibians worldwide, such as Mi et al. (2023) with 5403 species and Holt et al. (2013) with 6110 species, this might be due to our decision to exclusively use data from individuals deposited in museums and reported in GBIF. By making this decision, we aimed to reduce the overestimation of species distribution (species co‐omission) related to the use of distribution polygons, such as those from the IUCN (Boitani et al. 2011), and to avoid potential identification bias by not including citizen science data deposited in GBIF (Girardello et al. 2019). Nevertheless, our study includes more than 55% of species for almost all families across all realms worldwide, and, in terms of absolute percentage, our representativeness is only 7.4% lower than that of other recent global studies on amphibians (i.e., Mi et al. 2023). Therefore, we consider our dataset to be representative enough to help to understand amphibian distribution gaps globally.

We detected a general disparity between the number of records for each realm. The Nearctic, Neotropical, and Australasian realms together accounted for the majority of records, comprising 86.8% of the total. This observation partially agrees with the results of Hughes et al. (2021), who found that the Nearctic, Australasia, and Western Europe were the best represented regions in terms of number of records. Martin et al. (2012) mapped 2573 terrestrial study areas for work in ecology and found similar patterns, with most of the study areas found in the Nearctic, Western Europe, Neotropic, Southeastern Australia, and Oceania. In our study, the lowest number of records was for the Palearctic realm, comprising approximately 2.7% of the total records. Martin et al. (2012) and Hughes et al. (2021) both noted a high concentration of records for different taxonomic groups and study sites in Western Europe. In contrast, except for Japan, records in Asia and Eastern Europe are relatively low. This pattern is evident in our record distribution map (Figure 1). Authors such as Beck et al. (2014) and Ivanova and Shashkov (2021) reported geographical biases in GBIF records, suggesting that these biases may be related to the unequal distribution of research resources among countries, limiting their contributions to the databases GBIF relies on. When comparing data from the World Bank on “Research and Development” investment, it is evident that within the Palearctic realm, countries with higher GDP investments correspond to areas with more records (i.e., Western Europe, the Scandinavian Peninsula, Turkey, China, and Japan) (World Bank Open Data 2024). Interestingly, the World Bank data also show that the Afrotropical, Indo‐Malayan, and Neotropical realms have even lower GDP investments in Research and Development than several Palearctic countries, which would suggest a lower contribution in the number of records. However, Hughes et al. (2021) found a relationship between high‐record points for different taxonomic groups and maritime and road routes, indicating that realms such as the Nearctic influence the number of records in the Neotropical region, while Western Europe and the Australasian realm influence the Afrotropical and Indo‐Malayan realms. The high number of records in the Neotropical realm may be attributable to its high amphibian species richness, considered the realm with a greater potential for new species descriptions and a continuous rate of new species description (Frost 2024; Moura and Jetz 2021; Vera Candioti et al. 2023). Consequently, the record density in this realm may be a reflection of its high species number.

The Nearctic and Australasian realms had the lowest percentages of data‐gap areas and the highest proportions of well‐sampled and under‐sampled areas. In the other realms, the percentages of data‐gap areas were greater than the combined percentage of well‐sampled and under‐sampled areas. Our observations on sampling completeness levels between realms are similar to those found by Girardello et al. (2019) for butterflies, who observed the same completeness patterns, with North America, Australia, and Western Europe being the regions with the highest butterfly sampling completeness, while South America, Africa, and Eastern Europe –Asia had the lowest levels of completeness, similar to our findings. The differences in sampling completeness levels between realms may be related to the previously reported bias in the locations where ecological studies are conducted (Boakes et al. 2010; Martin et al. 2012; Romo et al. 2006). Realms such as the Nearctic, Australasian, and the western portion of the Palearctic have a higher number of recurrent ecological study sites, where biodiversity inventories can be more consistent, leading to higher sampling completeness. In contrast, realms like the Neotropical, Afrotropical, Indo‐Malayan, and the eastern portion of the Palearctic have a relatively low number of recurrent study sites (Beck et al. 2013; Boakes et al. 2010; Martin et al. 2012), and factors such as social and security instability (Hanson 2018; Murillo‐Sandoval et al. 2021; Siddig 2019) limit the expansion of research areas. This contributes to increased knowledge gaps in the distribution of various biological groups, such as amphibians, and consequently to gaps in the completeness of biological inventories.

**FIGURE 4:**
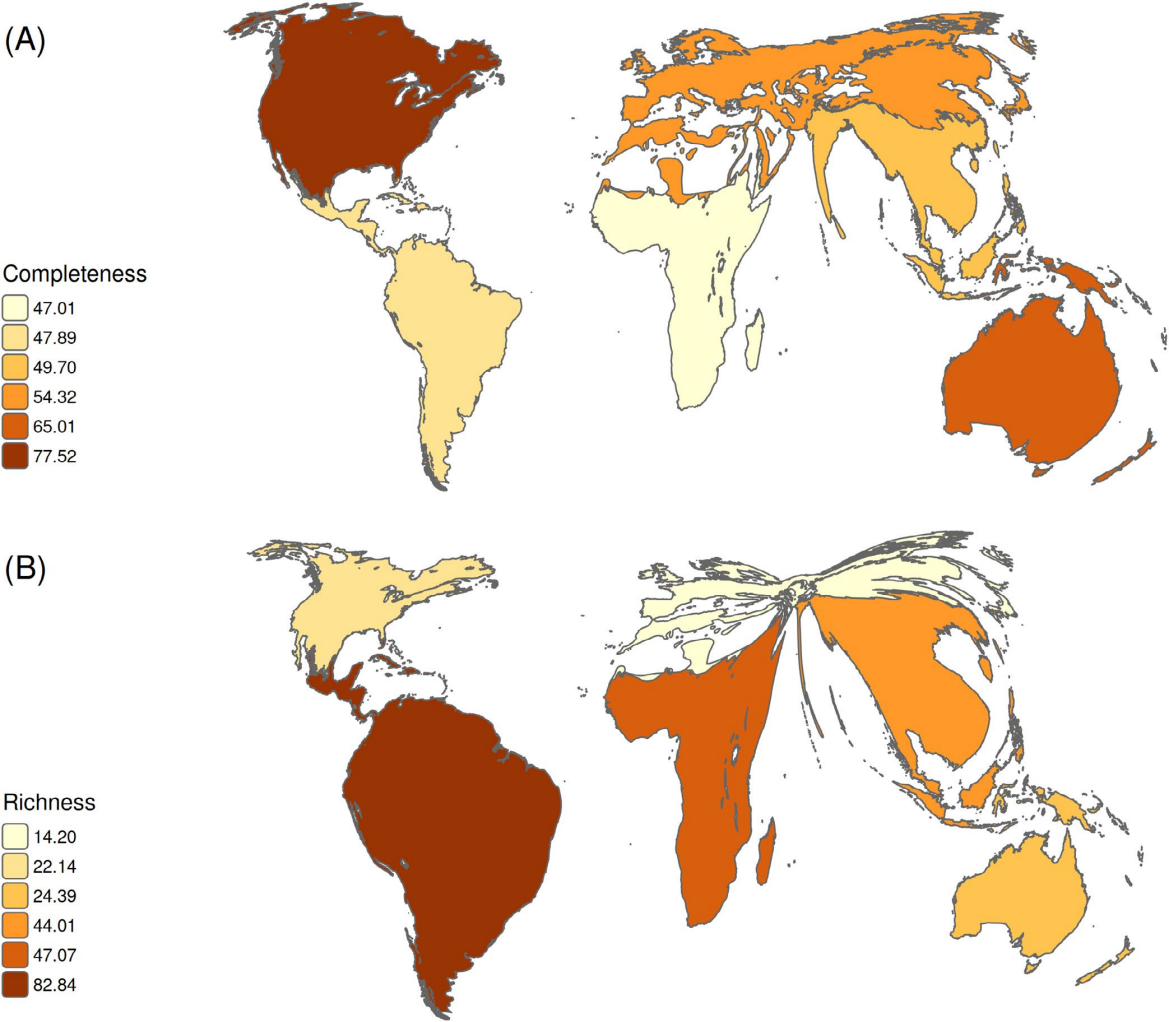
Area cartograms showing A. mean of sampling completeness per hexagon and B. mean of amphibian species richness per hexagon in each biogeographic realm. Larger polygons and warmer colors represent higher mean values per hexagon of sampling completeness or species richness in the realm.

An interesting inverse pattern is observed between sampling completeness and species richness. The Neotropical, Afrotropical, and Indo‐Malayan realms showed the highest number of species (Table 1). These realms, along with the Palearctic, exhibited the highest proportions of data gaps and under‐sampled areas (Table 2 and Figure 2). Distorted maps (area cartograms) based on sampling completeness and species richness (Figure 4) better illustrate this pattern. Moura and Jetz (2021), and Button and Borzée (2024) highlighted that most of the potential for discovering new vertebrate species lies in the Neotropical, Indo‐Malayan, and Afrotropical realms. Similarly, Vera Candioti et al. (2023) emphasized the gaps in ecological and natural history knowledge of the larval stages of anurans (the most numerous group of amphibians), especially in the Neotropical and Indo‐Malayan realms. Thus, the largest information gaps in distribution, natural history, and ecology occur precisely in the regions with the highest number of species. Given the transformation of ecosystems and global change affecting highly sensitive groups such as amphibians (Corn 2005), and considering that completeness can also be interpreted as the probability of adding new species with continued sampling (Button and Borzée 2024; Yang et al. 2013), it is possible that if research efforts are not increased in the under‐sampled and data gap areas of these realms, we may face the loss of species we are yet to discover.

The spatial overlap between well‐sampled, under‐sampled, and data‐gap areas with NPAs remained relatively low across biogeographic realms, staying below 35% (Table 3). Mi et al. (2023) noted that a significant proportion of amphibian species inhabited NPAs. Our results expand on this, suggesting that while many amphibians occur in NPAs, the limited overlap with well‐sampled areas points to gaps in understanding the full amphibian diversity within these protected regions. Campbell Grant et al. (2023) emphasized the need to determine the number and identity of amphibian species within NPAs, so we could underscore the importance of targeting sampling efforts in these areas as a critical first step in conservation planning. Furthermore, substantial under‐sampled and data‐gap areas outside NPAs indicate the potential for populations of conservation‐priority species or species yet to be described and should not be overlooked.

The spatial congruence between well‐sampled and under‐sampled areas with KBAs was relatively low (less than 35%). KBAs are identified based on diverse multitaxonomic criteria to prioritize conservation areas, aiming for complementarity among sites (IUCN 2016). These KBAs align with recent goals of the KMGBF established in 2022 (Kunming‐Montreal Global Biodiversity Framework 2022), which emphasizes conserving at least 30% of terrestrial and marine areas. KBAs should thus serve as the primary tool for defining new conservation areas based on biological criteria (IUCN 2016; Plumptre et al. 2024). The alignment of well‐sampled areas was proportionally higher than that of under‐sampled and data‐gap areas across all realms (Table 4), suggesting that KBAs primarily include areas where amphibian distributions are well‐documented. However, the low overlap indicates that not all well‐sampled areas are recognized as key conservation areas. Therefore, potential new KBAs may be supported by carefully examining under‐sampled and data‐gap areas. It is crucial to assess whether these regions support amphibian species and communities of significant biological and distributional importance, deserving inclusion in KBAs.

One of our objectives was to map areas with gaps in amphibian distribution data (under‐sampled and data‐gap areas) and provide spatial information on where to prioritize sampling efforts. In Supporting Information S2, we conduct a detailed review of under‐sampled and data‐gap regions at the biome level for each realm. However, as shown in Figure 3, it is critical to enhance sampling in the Neotropical, Indo‐Malayan, and Afrotropical realms, which encompass the highest amphibian species richness and the largest under‐sampled and data‐gap areas. These realms align with priority areas for the discovery of new vertebrate species as identified by Moura and Jetz (2021), and the Afrotropical and Indo‐Malayan realms have had the fewest biological sampling stations established (Martin et al. 2012).

Recently, Button and Borzée (2024) used amphibians and their level of sampling completeness to underscore the importance of defining priority areas for the discovery of new vertebrates, taking into account sampling completeness. In this straightforward exercise, they found patterns similar to those presented in our work. However, unlike their study, ours reviews how these levels of completeness are globally distributed and the level of congruence between well‐sampled, under‐sampled, and data‐gap areas with NPAs and KBAs. To our knowledge, our work is the first to attempt to quantify the sufficiency of sampling and the quality of amphibian distribution data on a global scale, utilizing data from accessible and open databases.

It is also important to emphasize the limitations of our study. Due to the taxonomic and geographic scale at which it was conducted, local applications may be challenging. The conservation by biome approach, presented in Supporting Information S2, should help enhance the applicability of our findings on a local scale by focusing on under‐sampled or data‐gap biomes within each domain rather than increasing sampling across entire hexagons. While GBIF offers a theoretically better alternative with fewer co‐omission errors compared to occurrence data from other sources, it is still imperfect. In using these data, we applied curation steps to reduce potential biases and used a completeness estimation method designed to mitigate bias from unequal sampling and heterogeneous data sources. However, due to our limited access to specimens and the fact that this falls beyond the scope of our study, we must rely on the proper identification of specimens deposited in museums. Additionally, given the scale used in the hexagons, we may have underestimated areas with information gaps. We hope this work serves as a starting point for directing research efforts both within and outside NPAs and KBAs, contributing to a better understanding of amphibian distribution based on specimen records at a global scale. We also anticipate that these data will improve KBAs and guide the development of new NPAs in line with international conservation goals.

Our study underscores that, for amphibians, much of the distribution information we have is incomplete, with larger gaps in some realms than in others. Given the challenges biodiversity faces from global change and human actions, rapid responses to understand species distribution and amphibian community structure are crucial. Only by understanding these aspects we should be able to devise effective conservation plans that align with political actions and global conservation initiatives, such as establishing NPAs and delineating KBAs. To quote Edward O. Wilson: “Biodiversity research requires more boots on the ground.” (Wilson 2017). Based on our findings, amphibian sampling efforts should particularly focus on the Indo‐Malayan, Afrotropical, and Neotropical realms, as well as the Asian portion of the Palearctic. These areas exhibit lower sampling completeness than other realms and have the potential to yield additional species for existing inventories.

## Author Contributions

**Jorge Mario Herrera‐Lopera:** conceptualization (equal), data curation (equal), formal analysis (equal), investigation (equal), methodology (equal), writing – original draft (equal), writing – review and editing (equal). **Mirco Solé:** conceptualization (equal), formal analysis (equal), funding acquisition (equal), investigation (equal), methodology (equal), supervision (equal), writing – original draft (equal), writing – review and editing (equal). **Carlos A. Cultid‐Medina:** conceptualization (equal), formal analysis (equal), investigation (equal), methodology (equal), supervision (equal), writing – original draft (equal), writing – review and editing (equal).

## Conflicts of Interest

The authors declare no conflicts of interest.

## Supporting information


Appendix S1.

## Data Availability

The databases and R language code used for the analysis of this work are available in DRYAD at http://datadryad.org/stash/share/N‐QvzsrurL2I9oEJPyriBVJWwFJWe2U4a82kklxKnok or by contacting the authors. To get processed spatial files, please contact the authors.
