# Supplementary material for: Mapping the Missing: Assessing Amphibian Sampling Completeness and Overlap With Global Protected Areas
Source: Ecol Evol. 2025 May 5;15(5):e71137. doi: 10.1002/ece3.71137 (PMC12052474; doi:10.1002/ece3.71137)
Supplement: Supplementary file 1 — Appendix S1. [file ECE3-15-e71137-s001.docx]

**Mapping the missing: assessing amphibian sampling completeness and overlap with global protected areas**

Jorge Mario Herrera-Lopera ^1, 2, 3^, Mirco Solé ^1, 2, 4, 5^, Carlos A. Cultid-Medina ^6, 7^

^1^ Programa de Pós-Graduação em Ecologia e Conservação da Biodiversidade, Universidade Estadual de Santa Cruz, Ilhéus 45662-900, Brazil.

^2^ Tropical Herpetology Lab, Departamento de Ciências Biológicas, Universidade Estadual de Santa Cruz, Ilhéus 45662-900, Brazil.

^3^ Grupo de Investigación en Biodiversidad y Recursos Naturales (BIONAT), Semillero de investigación en Biodiversidad y Conservación de Paisajes Urbanos (OIKOS), Universidad de Caldas, Manizales 170001, Colombia.

^4^ Departamento de Ciências Biológicas, Universidade Estadual de Santa Cruz (UESC), Ilhéus 45662-900, Brazil

^5^ Museum Koenig Bonn (ZFMK), Leibniz Institute for the Analysis of Biodiversity Change, Bonn 53113, Germany. Email: [*msole@uesc.br*](mailto:msole@uesc.br)

^6^ Red de Diversidad Biológica del Occidente Mexicano Instituto de Ecología, A. C., Centro Regional del Bajío Pátzcuaro 61600, México. Email: [*carlos.cultid@inecol.mx*](mailto:carlos.cultid@inecol.mx)

^7^ CONAHCYT, Ciudad de México 03940, México.

**Correspondence:**

Jorge Mario Herrera-Lopera, Programa de Pós-Graduação em Ecologia e Conservação da Biodiversidade, Universidade Estadual de Santa Cruz, Ilhéus, Bahia, Brazil. Email*:* [*mario.herreralopera@gmail.com*](mailto:mario.herreralopera@gmail.com) *–* [*jmhlopera@uesc.br*](mailto:jmhlopera@uesc.br)

Supporting Information S1: Selecting biogeographical proposal

We considered two biogeographical frameworks: Olson et al. (2001) modified by Dinerstein et al. (2017), and the framework proposed by Holt et al. (2013). To assess the suitability of these frameworks for amphibian distribution data, we took two approaches: (i) we created clusters using the Jaccard dissimilarity index (Carvalho et al. 2013) with a dissimilarity cutoff of 0.75, such that if two realms in a framework had a dissimilarity less than 0.75, we would deem it unsuitable; and (ii) using 2° wide hexagons as subsamples for each realm, we conducted an ANOSIM to determine if the variation within realms of each framework was smaller or greater than the variation between realms. If the variation between realms was smaller than the variation within realms, we would consider the framework unsuitable for our data.


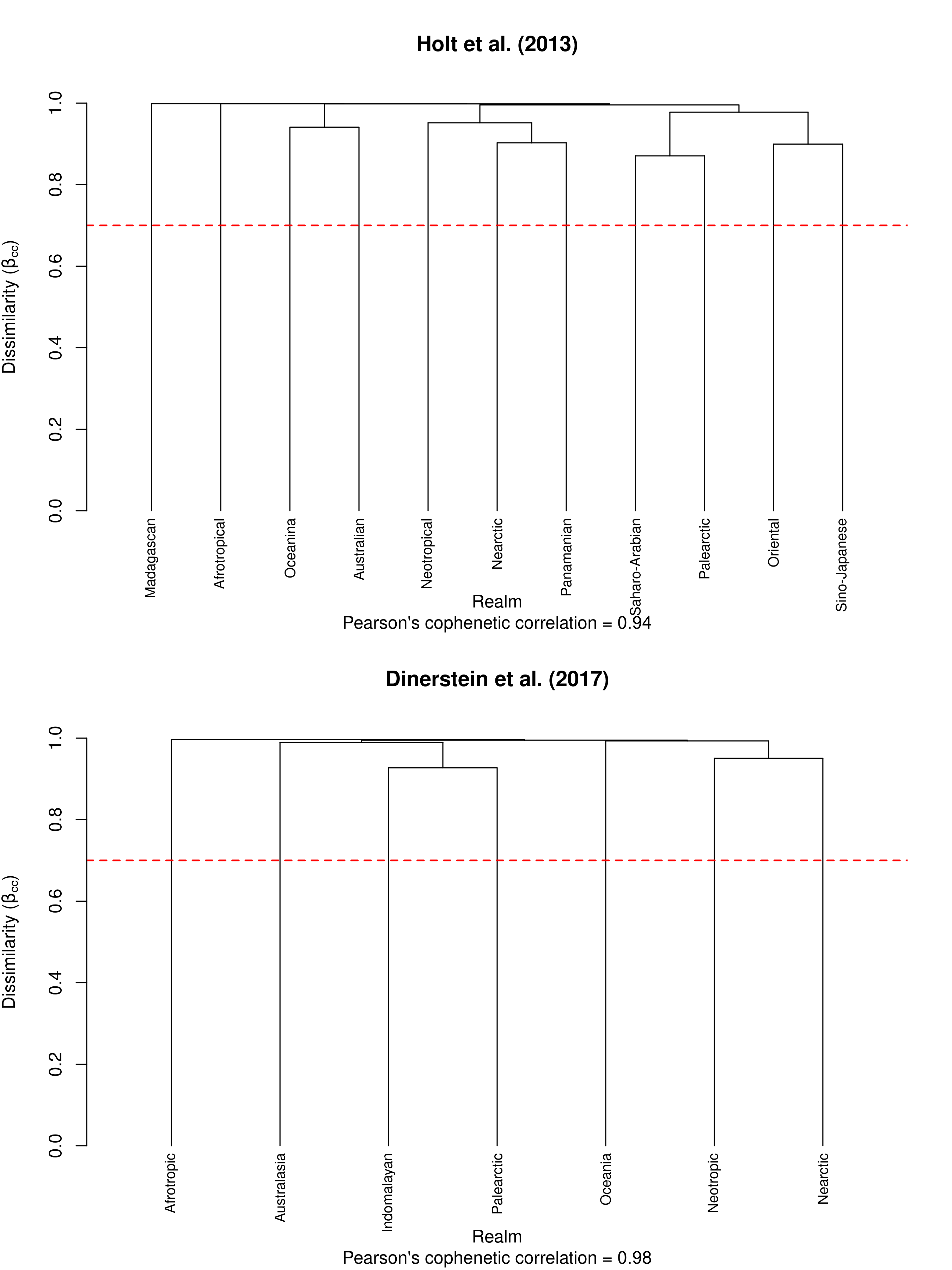
**Figure S1**. Clustering of realms according to Holt et al. (2013) and Dinerstein et al. (2017). Distance is measured using the Jaccard dissimilarity index. The line represents a dissimilarity cutoff of 75% (0.75).

For the first approach, we found that both Holt et al. (2013) realms and Dinerstein et al. (2017) realms may be suitable for separating amphibian distribution data at a global level (Figure S1). Therefore, based on the clustering results, either approach would be equally valid.

For the second approach, we found R = 0.84, P < 0.00 for Holt et al. (2013), and R = 0.65, P < 0.00 for Dinerstein et al. (2017). Positive values in the R statistic indicate greater variation between realms than within them, suggesting both biogeographical frameworks are suitable for analyzing global amphibian distribution, as per ANOSIM results.

Given both frameworks seemed equally valid for segmenting global amphibian distribution, we opted for Dinerstein et al. (2017)approach because it allows for the analysis of information gaps at the biome level. Although a biome-level analysis is not one of our primary objectives, we included it as an additional analysis in Supporting Information S2

Supporting Information S2: well-sampled, under-sampled, and data-gap areas across biomes and realms

**Afrotropic realm**

*Well-sampled areas*: 2% of Afrotropical realm were well-sampled. These areas were located mainly in the southern and central-western regions, particularly in South Africa, and in smaller areas in Botswana, Namibia, Tanzania, Democratic Republic of Congo, Uganda and Ethiopia. The biomes with well-sampled hexagons were: Tropical & Subtropical Grasslands, Savannas & Shrublands, Mediterranean Forests, Woodlands & Scrub, Deserts & Xeric Shrublands and in smaller quantities Tropical & Subtropical Moist Broadleaf Forests and Montane Grasslands & Shrublands. The other biomes did not have any areas that could be classified as well sampled within this realm (Figure S2).


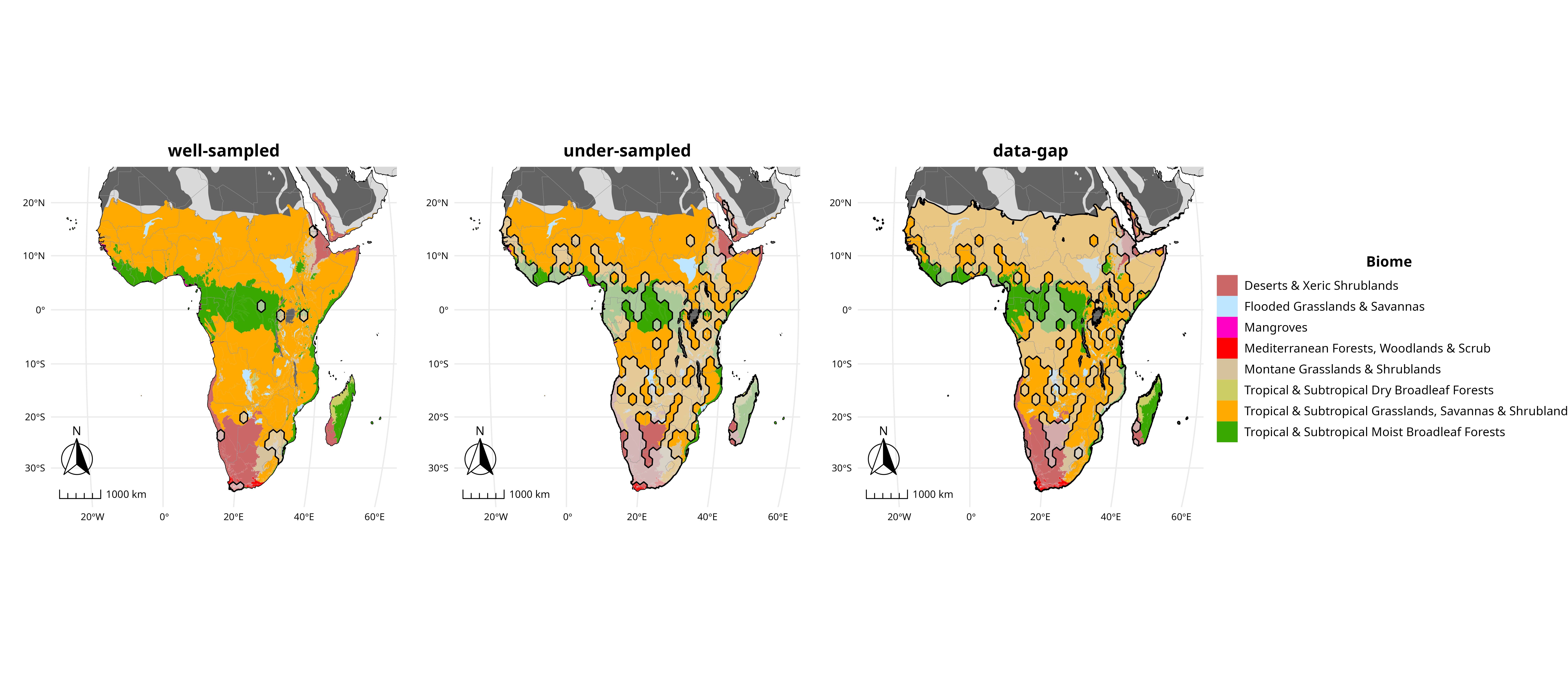
**Figure S2**. Well-sampled, under-sampled and data-gap areas in the biomes of Afrotropic realm. Dark gray areas indicate regions excluded because no amphibian species distributions are expected there according to the (IUCN 2024) spatial data. Light gray areas indicate the area belonging to other realms.

*Under-sampled areas*: 42% of the Afrotropical realm is under-sampled. These areas are primarily located in the southern, west-central, and east-central regions. Under-sampled areas exist in every country within the realm, except for Mauritania, Mali, and Chad. Most of Madagascar and the part of the realm within the Arabian Peninsula are also under-sampled. In terms of biomes, most under-sampled areas are found within the Tropical & Subtropical Grasslands, Savannas & Shrublands, Deserts & Xeric Shrublands, Tropical & Subtropical Dry Broadleaf Forests, and Tropical & Subtropical Moist Broadleaf Forests biomes. To a lesser extent, under-sampled areas are also present in the Mediterranean Forests, Woodlands & Scrub, Flooded Grasslands & Savannas and Montane Grasslands & Shrublands biomes (Figure S2).

*Data-gap areas*: 55% of the Afrotropical realm lacked sufficient data to estimate sampling completeness (i.e., had deficient-data). These deficient-data areas were mainly located in the northern and central regions of the realm and were present in every country within it. At the biome level, all except Tropical & Subtropical Dry Broadleaf Forests and Mediterranean Forests, Woodlands & Scrub had large areas with deficient-data. Notably, the Mangroves and Flooded Grasslands & Savannas biomes had the majority of their area with deficient-data. Additionally, the Tropical & Subtropical Grasslands, Savannas & Shrublands and Tropical & Subtropical Moist Broadleaf Forests biomes had extensive areas with deficient-data (Figure S2).

**Australasia realm**

*Well-sampled areas*: 21% of the Australasian realm was found to be well-sampled for amphibian distribution. The majority of these well-sampled areas were in Australia, with smaller portions in Papua New Guinea and Indonesia. In Australia, most well-sampled zones were in the southeast, Tasmania, north, and central regions, with fewer well-sampled areas in the southeast and south-central regions. At biome level, the Temperate Broadleaf & Mixed Forests, Tropical & Subtropical Grasslands, Savannas & Shrublands, and Mediterranean Forests, Woodlands & Scrub biomes were well-sampled. To a lesser extent, the Temperate Grasslands, Savannas & Shrublands, Tropical & Subtropical Moist Broadleaf Forests, and Deserts & Xeric Shrublands biomes were also well-sampled. Nearly all well-sampled biomes were within Australia. In Papua New Guinea and Indonesia, portions of the Montane Grasslands & Shrublands and Tropical & Subtropical Moist Broadleaf Forests biomes were also well-sampled (Figure S3).

*Under-sampled areas*: 42% of the Australasian realm was found to be under-sampled. Most of these under-sampled areas were located in Papua New Guinea, Indonesia, and the northern, east-central, and southwestern regions of Australia. In terms of biomes, the Temperate Grasslands, Savannas & Shrublands, Tropical & Subtropical Grasslands, Savannas & Shrublands, Tropical & Subtropical Moist Broadleaf Forests, and Mediterranean Forests, Woodlands & Scrub biomes had the highest proportion of under-sampled areas (Figure S3).


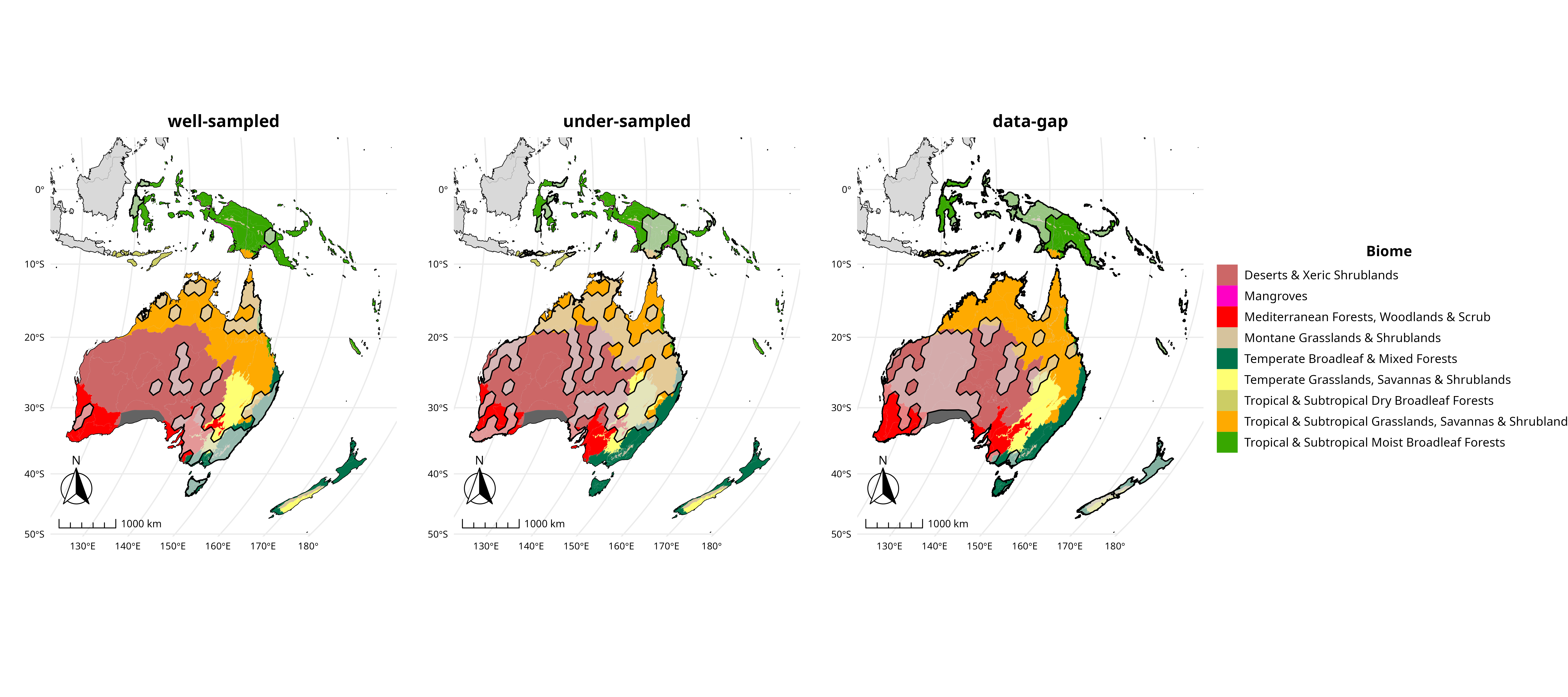
**Figure S3**. Well-sampled, under-sampled and data-gap areas in the biomes of Australasian realm. Dark gray areas indicate regions excluded because no amphibian species distributions are expected there according to the (IUCN 2024) spatial data. Light gray areas indicate the area belonging to other realms.

*Data-gap areas*: The Australasian realm had 37% of its area categorized as deficient-data. Most of these areas were located in New Zealand, Indonesia, and the western and central regions of Australia. In terms of biomes, the Tropical & Subtropical Dry Broadleaf Forests, Mangroves, Tropical & Subtropical Moist Broadleaf Forests, Montane Grasslands & Shrublands, and Deserts & Xeric Shrublands had the largest proportion of their areas classified as deficient-data (Figure S3).

**Indo-Malayan realm**

*Well-sampled areas*: Well-sampled areas accounted for 1% of the total area of the Indo-malayan realm. These areas were located in Taiwan, the Philippines, and Myanmar, covering small portions of the Tropical & Subtropical Moist Broadleaf Forests and Tropical & Subtropical Dry Broadleaf Forests biomes (Figure S4).

*Under-sampled areas*: Under-sampled areas accounted for 38% of the Indo-malayan realm. The majority of this area was found in the Indochinese Peninsula and the western portion of India. Additionally, other under-sampled areas were identified in Malaysia, Indonesia, Sri Lanka, China, and the Philippines. In terms of biomes, most of the undar-sampled areas were located in the Tropical & Subtropical Moist Broadleaf Forests, Tropical & Subtropical Dry Broadleaf Forests, and Tropical & Subtropical Grasslands, Savannas & Shrublands (Figure S4).


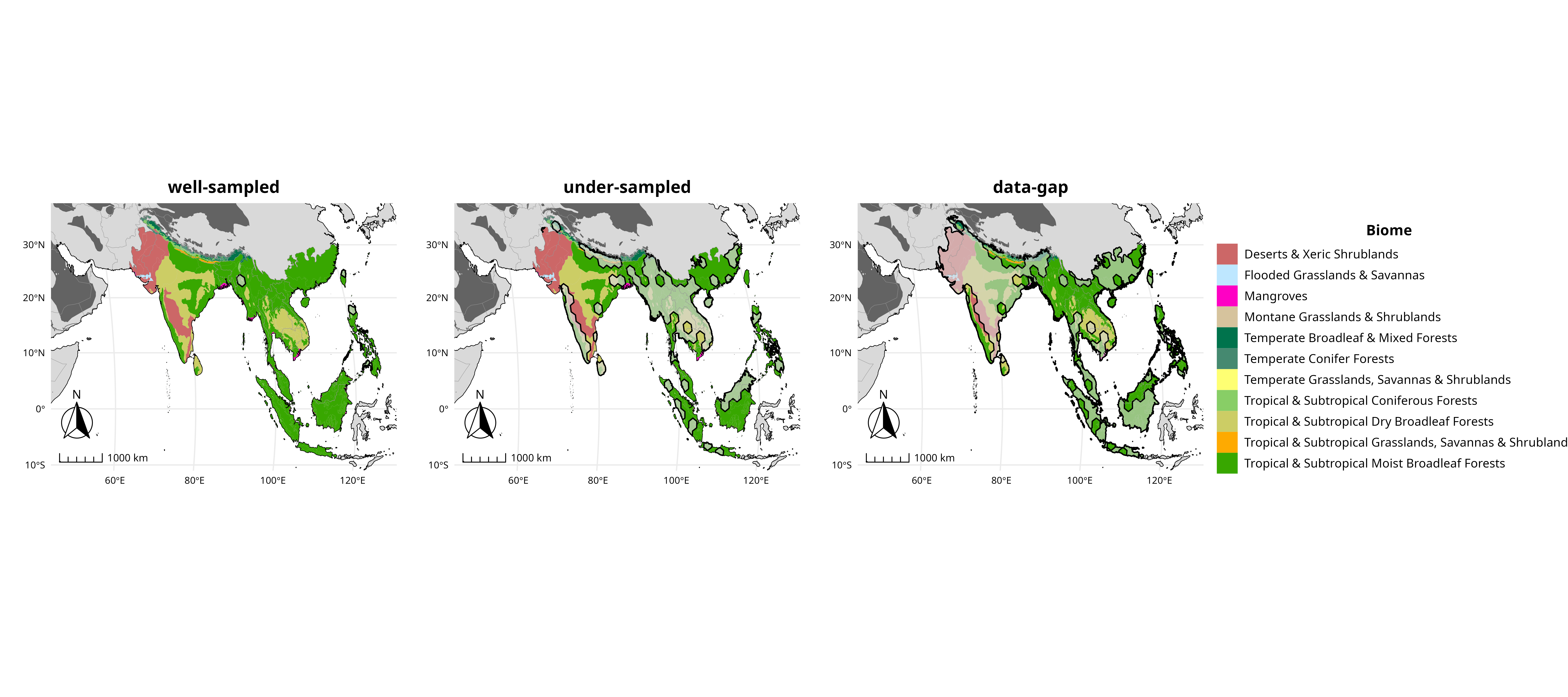
**Figure S4**. Well-sampled, under-sampled and data-gap areas in the biomes of Indo-malayan realm. Dark gray areas indicate regions excluded because no amphibian species distributions are expected there according to the (IUCN 2024) spatial data. Light gray areas indicate the area belonging to other realms.

*Data-gap areas*: Deficient-data areas covered 60% of the Indo-malayan realm. These areas were predominantly located in the Indian subcontinent, China, Indonesia, and Thailand. All biomes had areas with deficient-data. However, the Mangroves, Flooded Grasslands & Savannas, and Tropical & Subtropical Dry Broadleaf Forests biomes stand out, with the majority of their area within the realm classified as data-gap areas (Figure S4).

**Neartic realm**

*Well-sampled areas*: Well-sampled areas comprised 40% of the entire Neartic realm. The majority of these areas were located in the United States, with smaller portions in Mexico and Canada. Significant portions of all biomes were classified as well-sampled, although the Boreal Forests/Taiga and Tropical & Subtropical Coniferous Forests biomes had comparatively smaller proportions (Figure S5).

*Under-sampled areas*: Under-sampled areas comprised 26% of the total area of the Nearctic realm. These areas were predominantly located in the central-northern regions of the United States, southern Canada, and northern Mexico. The majority of under-sampled areas were concentrated in the Temperate Broadleaf & Mixed Forests, Temperate Grasslands, Savannas & Shrublands, and Deserts & Xeric Shrublands biomes (Figure S5).


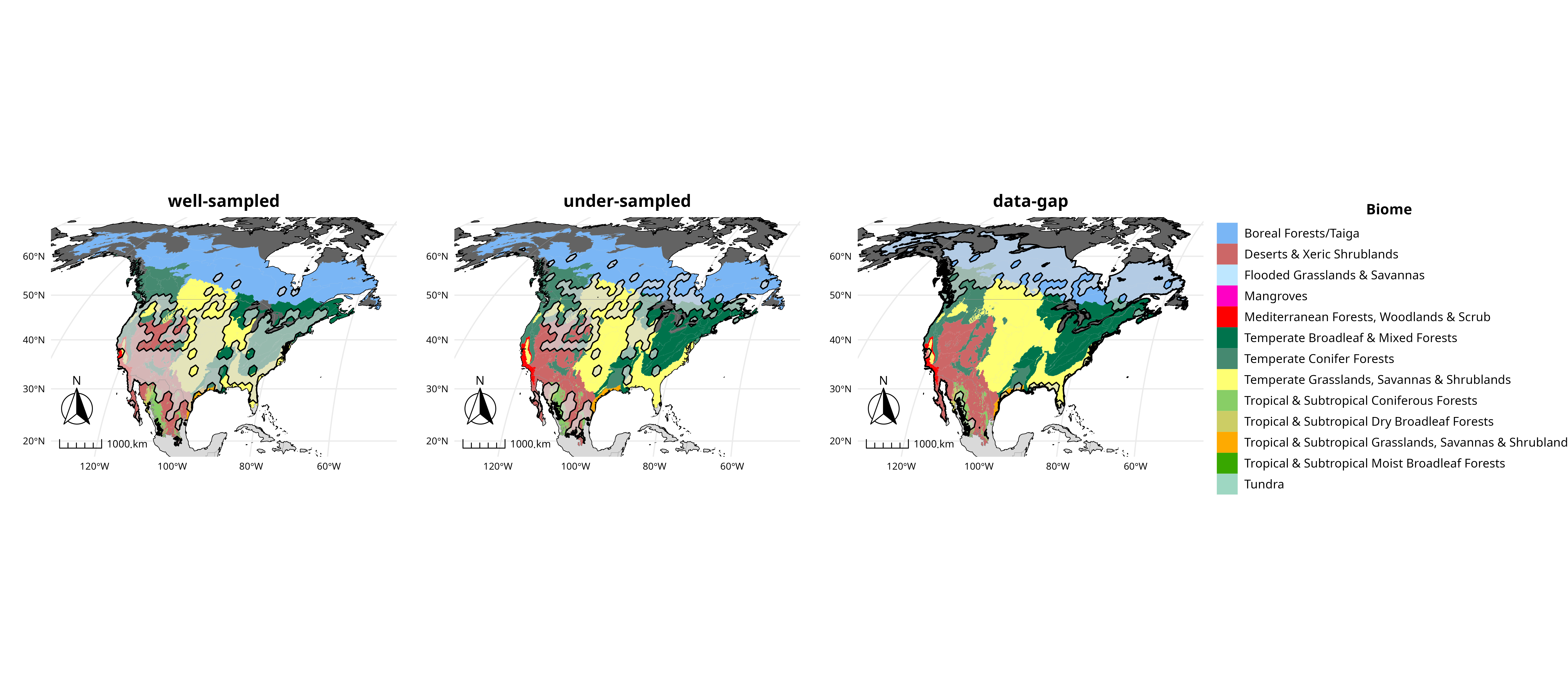
**Figure S5**. Well-sampled, under-sampled and data-gap areas in the biomes of Neartic realm. Dark gray areas indicate regions excluded because no amphibian species distributions are expected there according to the (IUCN 2024) spatial data. Light gray areas indicate the area belonging to other realms.

*Data-gap areas*: Data-gap areas accounted for 35% of the Nearctic realm. These areas were mainly distributed in Canada, the southern United States, and the western portion of northern Mexico. Regarding biomes, nearly all data-gap areas were concentrated in the Tropical & Subtropical Coniferous Forests, Temperate Broadleaf & Mixed Forests, Tropical & Subtropical Moist Broadleaf Forests, as well as Temperate Grasslands, Savannas & Shrublands, and to a lesser extent, Tropical & Subtropical Grasslands, Savannas & Shrublands (Figure S5).

**Neotropic realm**

*Well-sampled areas*: Well-sampled areas covered 7% of the total area of the Neotropical realm. These areas were located in the Andean region, specifically in the northern Andes and the southern portion between Argentina and Chile, as well as in southeastern Brazil, and in Central America, including Guatemala, Belize, Costa Rica, and Panama. At the biome level, most well-sampled areas were in the Tropical & Subtropical Moist Broadleaf Forests, Tropical & Subtropical Grasslands, Savannas & Shrublands, and Tropical & Subtropical Dry Broadleaf Forests. There were smaller proportions in Mangroves and Temperate Broadleaf & Mixed Forests (Figure S6).

*Under-sampled areas*: Under-sampled areas covered 48% of the total Neotropical realm. These areas were present in all countries within the realm except El Salvador and Guatemala. Furthermore, under-sampled areas were found in all biomes within the realm, with smaller proportions in the Tropical & Subtropical Dry Broadleaf Forests, Temperate Grasslands, Savannas & Shrublands, and Montane Grasslands & Shrublands biomes (Figure S6).


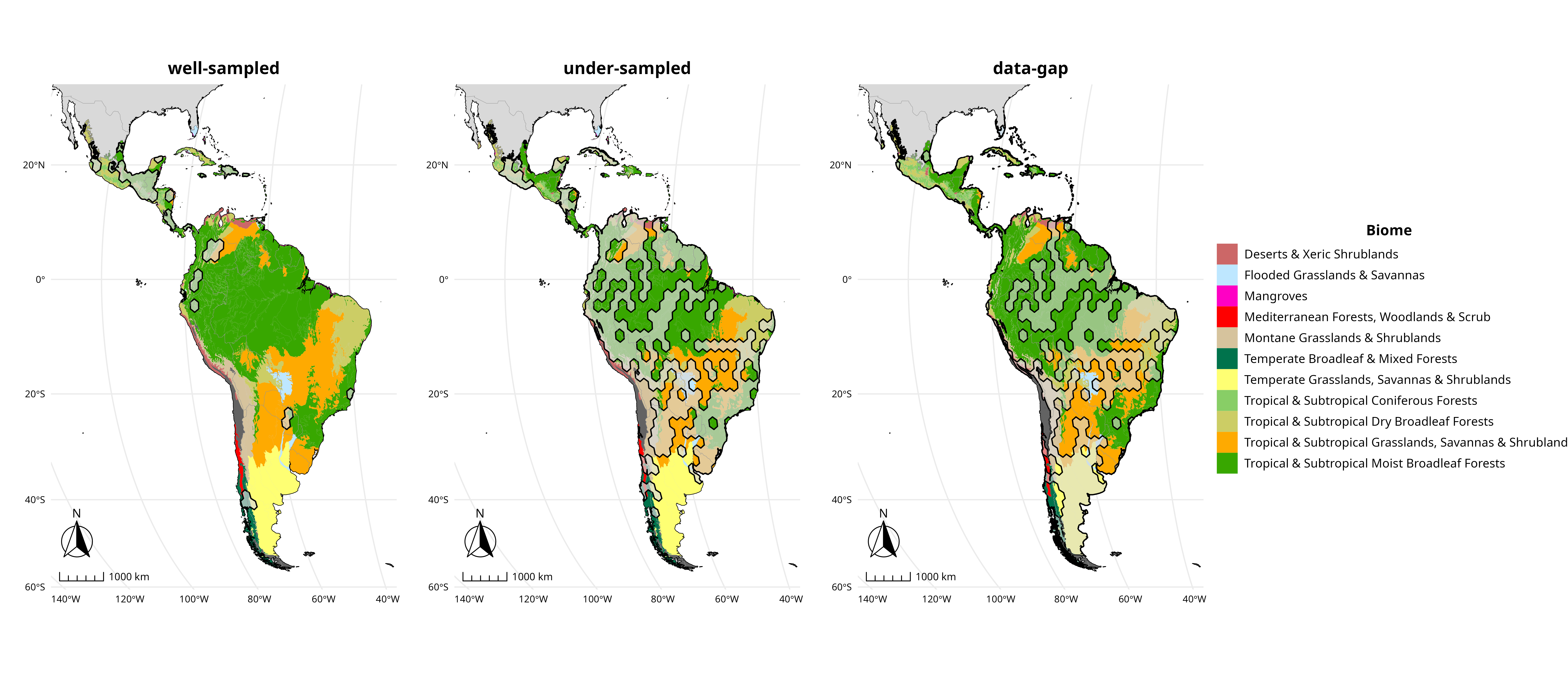
**Figure S6**. Well-sampled, under-sampled and data-gap areas in the biomes of Neotropic realm. Dark gray areas indicate regions excluded because no amphibian species distributions are expected there according to the (IUCN 2024) spatial data. Light gray areas indicate the area belonging to other realms.

*Data-gap areas*: Data-gap areas covered 45% of the total area of the Neotropical realm. The majority of these areas were located in South America, with some exceptions in Mexico and the Caribbean. In South America, data-gap areas were especially prevalent in the Amazon region, and in northern, northeastern, and western Brazil, as well as in Argentina. Most data-gap areas were found in the Tropical & Subtropical Moist Broadleaf Forests, Temperate Grasslands, Savannas & Shrublands, and Montane Grasslands & Shrublands biomes. To a lesser extent, they were also present in the Tropical & Subtropical Grasslands, Savannas & Shrublands, Tropical & Subtropical Dry Broadleaf Forests, and Deserts & Xeric Shrublands biomes (Figure S6).

**Paleartic realm**

*Well-sampled areas*: Well-sampled areas comprised 2% of the Palearctic realm. These areas were primarily located in the central-western region of Europe, the Scandinavian and Iberian Peninsulas, and Central Asia. Additionally, there were scattered well-sampled spots in the Balkans region and North Africa, particularly between Egypt and Israel. At the biome level, most well-sampled areas were found in the Temperate Broadleaf & Mixed Forests and Mediterranean Forests, Woodlands & Scrub biomes, with smaller proportions in the Deserts & Xeric Shrublands and Temperate Grasslands, Savannas & Shrublands biomes (Figure S7).


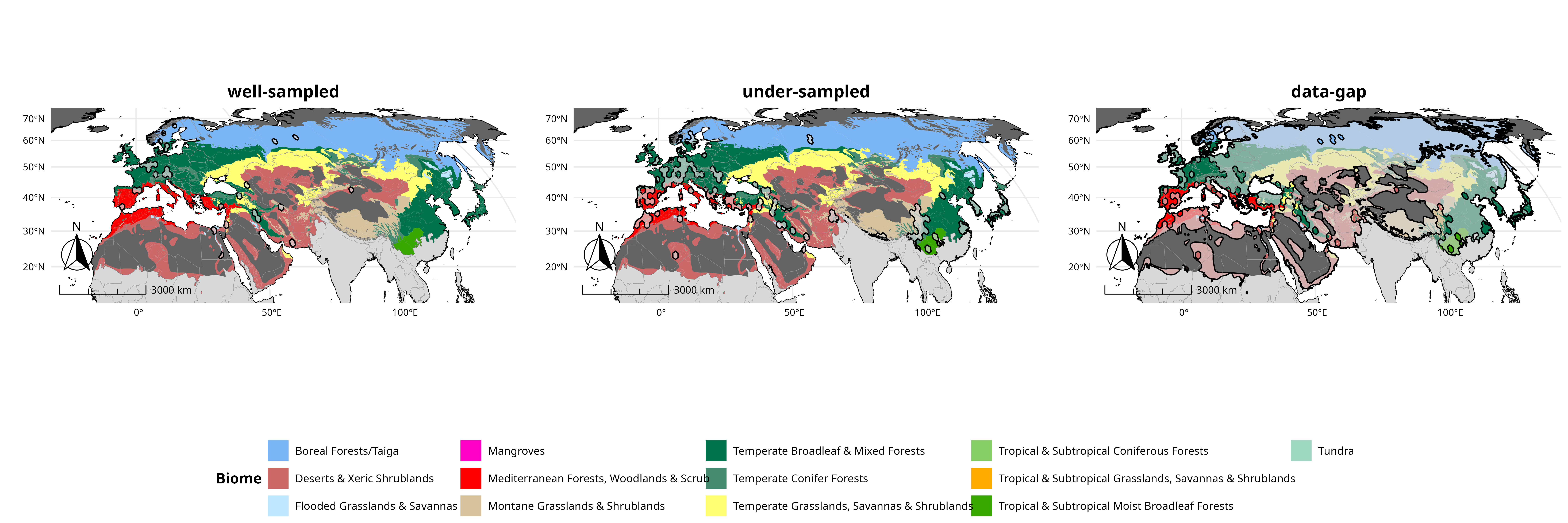
**Figure S7**. Well-sampled, under-sampled and data-gap areas in the biomes of Paleartic realm. Dark gray areas indicate regions excluded because no amphibian species distributions are expected there according to the (IUCN 2024) spatial data. Light gray areas indicate the area belonging to other realms.

*Under-sampled areas*: Under-sampled areas accounted for 10% of the total area of the Palearctic realm. These areas were primarily distributed in Western Europe, particularly in the Iberian Peninsula, the Mediterranean region, and the Scandinavian Peninsula. Under-sampled areas were also found in the western part of Asia, China, the Korean Peninsula, and Japan. At the biome level, the main under-sampled areas were in the Mediterranean Forests, Woodlands & Scrub, Montane Grasslands & Shrublands, and Temperate Broadleaf & Mixed Forests biomes, with smaller proportions in the Temperate Grasslands, Savannas & Shrublands, and Boreal Forests/Taiga biomes (Figure S7).

*Data-gap areas*: Data-gap areas covered 88% of the total area of the realm. Consequently, these areas spanned nearly the entire realm, with the exception of Western Europe and East Asia (the Korean Peninsula, Japan, and China). Given their extensive distribution, data-gap areas were present in all biomes, with smaller proportions found in the Temperate Broadleaf & Mixed Forests and Mediterranean Forests, Woodlands & Scrub biomes (Figure S7).

Supporting Information S3: Choosing hexagon size

To select the hexagon size (resolution) within the analysis, (i) we calculated the effect size of the hexagon on species richness and sampling completeness using omega squared (ω^2^) as the effect size measure (Kroes and Finley 2023), chosen for its low sensitivity to sample size differences and violation of the equal variances assumption. Additionally, (ii) we compared the 95% confidence intervals for species richness and sampling completeness across hexagon sizes. For both approaches, hexagon sizes ranged from 1° to 3.5°, with increments of 0.5°.

In terms of effect size, we determined that both species richness and completeness were influenced by hexagon size to a degree of less than 1% ( ω^2^ richness: 0.007, ω^2^ completeness: 0.002). Thus, objectively, the hexagon size used exerts minimal influence on sampling completeness and species richness. In terms of comparing confidence intervals for species richness and sampling completeness across different hexagon sizes, we observed an increasing trend in both metrics as hexagon size increases (Figure S8), consistent with findings from other studies (Bosco et al. 2022; Victorero et al. 2023). it's important to note that increasing hexagon size also increases errors of co-omission, where species presence is assumed within an area lacking confirming evidence. Hexagon sizes of 2° show significantly higher values of richness and completeness compared to 1° and 1.5° hexagons, with completeness values statistically equivalent to those of hexagons up to 3°, making it a potentially suitable middle ground. Moreover, this resolution aligns with the used by Holt et al. (2013). Given these considerations and the effect size being less than 1%, we opted for a 2° hexagon size to optimize richness and completeness while mitigating the risk of excessive co-omission errors.


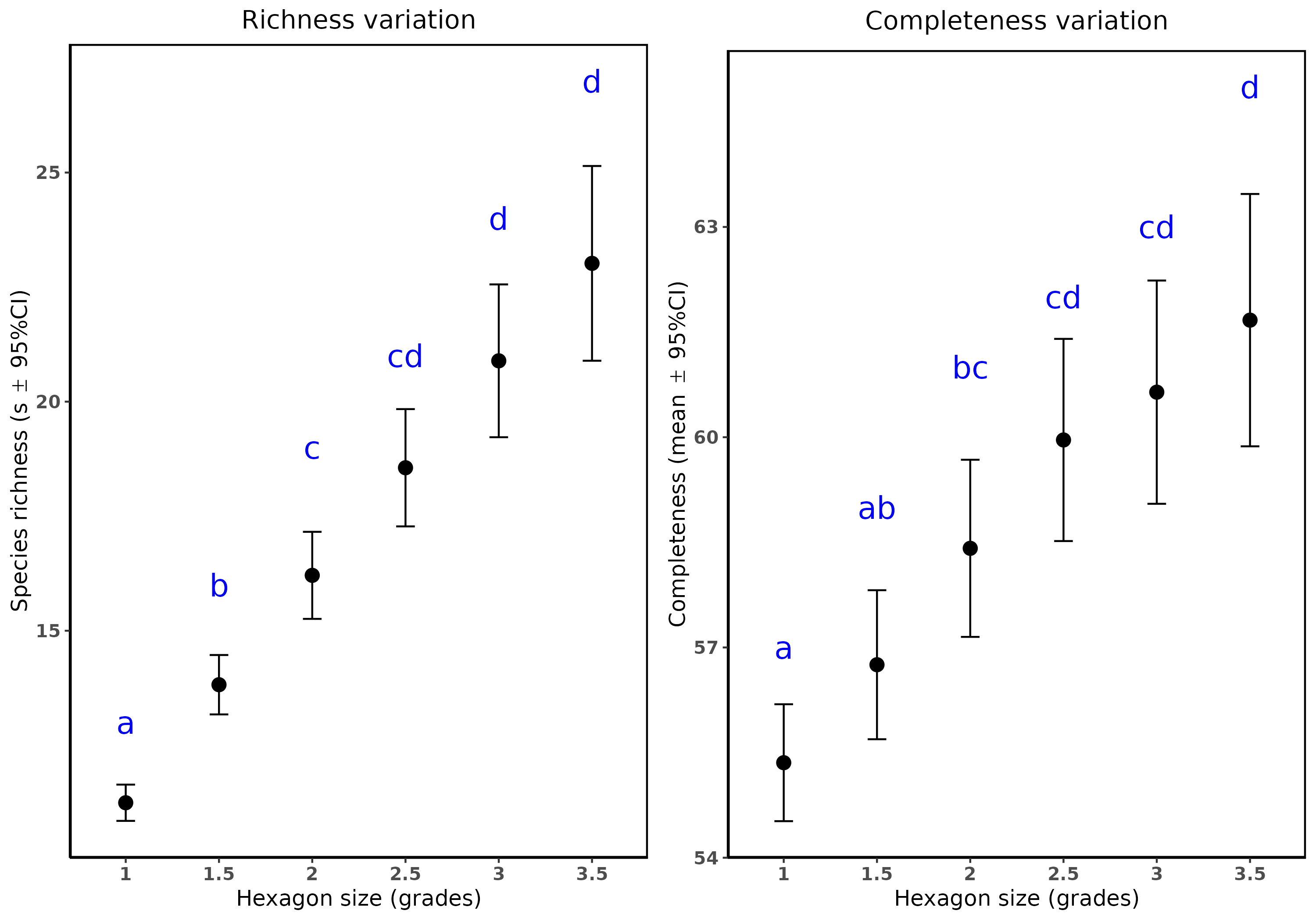
**Figure S8**. Variations in A. Species richness and B. Sampling completeness using different resolutions (hexagon sizes). Bars indicate 95% confidence intervals.

Supporting Information S4: Sampling completeness and completeness slope.

Completeness (Figure S9A) and accumulation curve slope (Figure S9B) observed at the global level. The accumulation curve slope was used as the basis for classifying well-sampled and under-sampled areas. Following a modified approach based on the criteria of Yang et al. 2013 and Girardello et al. 2019, we defined three categories: (i) “well-sampled” (slope ≤ 0.05), (ii) “under-sampled” (slope > 0.05), and (iii) “data-gap,” referring to areas where species records are expected according to (IUCN 2024) spatial data but where sampling completeness could not be assessed due to insufficient records (i.e., record-to-species ratio ≤ 1).


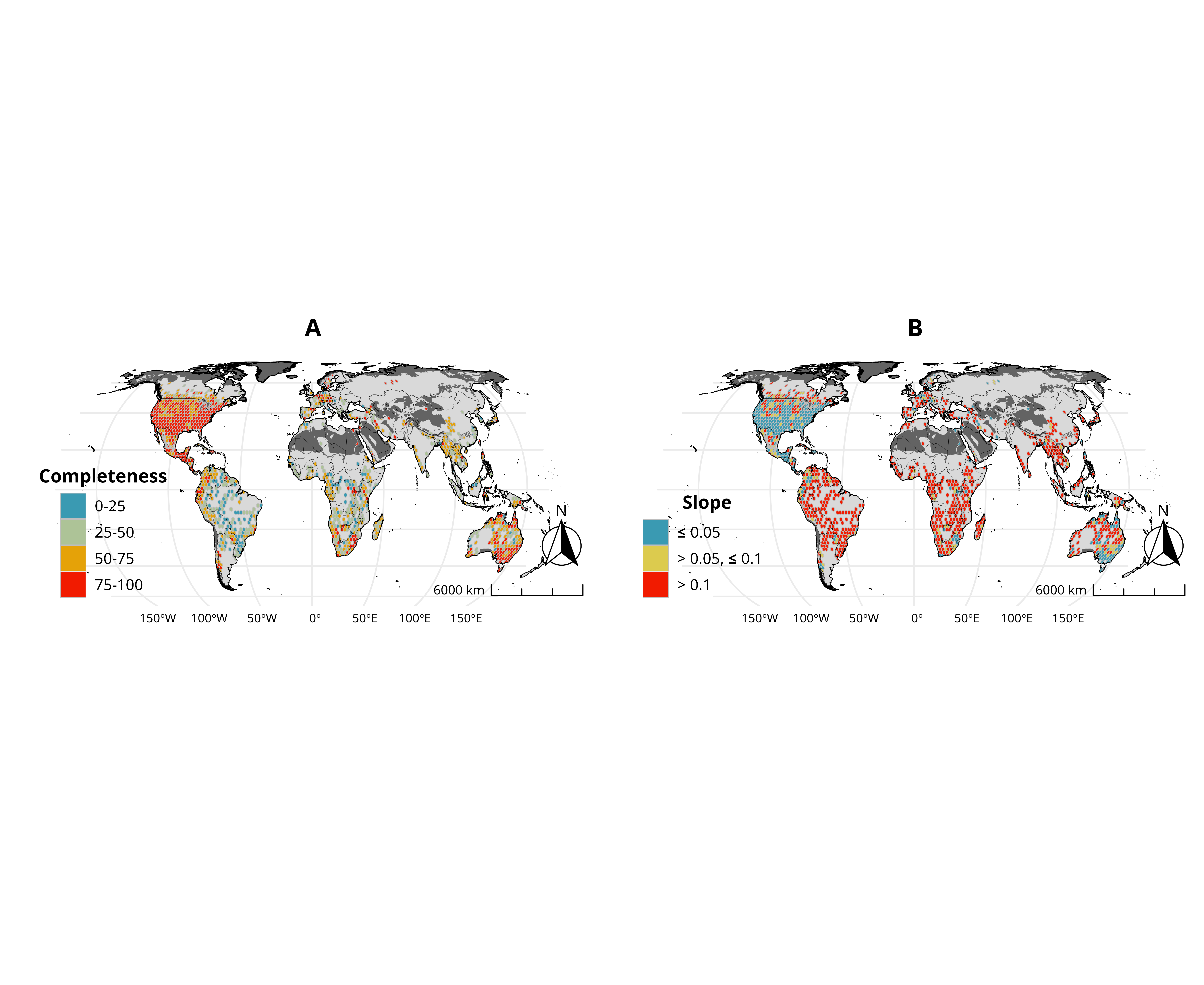
**Figure S9**. A. Sampling completeness estimation, B. Accumulation curve slope

**References**

Bosco NS, Prasniewski VM, Santos JP, et al (2022) Scale affects the understanding of biases on the spatial knowledge of Atlantic Forest primates. Perspect Ecol Conserv 20:338–345. https://doi.org/10.1016/j.pecon.2022.08.002

Carvalho JC, Cardoso P, Borges PAV, et al (2013) Measuring fractions of beta diversity and their relationships to nestedness: a theoretical and empirical comparison of novel approaches. Oikos 122:825–834. https://doi.org/10.1111/j.1600-0706.2012.20980.x

Dinerstein E, Olson D, Joshi A, et al (2017) An Ecoregion-Based Approach to Protecting Half the Terrestrial Realm. BioScience 67:534–545. https://doi.org/10.1093/biosci/bix014

Girardello M, Chapman A, Dennis R, et al (2019) Gaps in butterfly inventory data: A global analysis. Biol Conserv 236:289–295. https://doi.org/10.1016/j.biocon.2019.05.053

Holt BG, Lessard J-P, Borregaard MK, et al (2013) An Update of Wallace’s Zoogeographic Regions of the World. Science 339:74–78. https://doi.org/10.1126/science.1228282

IUCN (2024) Spatial Data Download

Kroes ADA, Finley JR (2023) Demystifying omega squared: Practical guidance for effect size in common analysis of variance designs. Psychol Methods. https://doi.org/10.1037/met0000581

Olson DM, Dinerstein E, Wikramanayake ED, et al (2001) Terrestrial Ecoregions of the World: A New Map of Life on Earth. BioScience 51:933. https://doi.org/10.1641/0006-3568(2001)051[0933:TEOTWA]2.0.CO;2

Victorero L, Samadi S, O’Hara TD, et al (2023) Global benthic biogeographical regions and macroecological drivers for ophiuroids. Ecography 2023:e06627. https://doi.org/10.1111/ecog.06627

Yang W, Ma K, Kreft H (2013) Geographical sampling bias in a large distributional database and its effects on species richness–environment models. J Biogeogr 40:1415–1426. https://doi.org/10.1111/jbi.12108
